# Circulating miR-26a-1, miR-146a and miR-199a-1 are potential candidate biomarkers for acute myocardial infarction

**DOI:** 10.1186/s10020-019-0086-1

**Published:** 2019-05-15

**Authors:** Sheng Xue, Wenjie Zhu, Dacheng Liu, Zhe Su, Liwei Zhang, Qing Chang, Peifeng Li

**Affiliations:** 1 Institute for Translational Medicine, College of Medicine, Qingdao University, Qingdao, 266021 China; 2 Affiliated Hospital of Qingdao University. Qingdao University, Qingdao, 266003 China

**Keywords:** Circulating miRNA, Acute myocardial infarction, Percutaneous coronary intervention, Biomarker

## Abstract

**Background:**

Acute myocardial infarction (AMI) was considered to be one of the major causes of morbidity and mortality worldwide. In order to manage the acute myocardial infarction outbreaks, accurate biomarkers for risk prediction are needed. Circulating microRNAs (miRNAs) may act as diagnostic and prognostic biomarkers for cardiovascular events.

**Methods:**

This study aimed to determine the possibility of circulating miRNAs used as biomarkers for AMI and their dynamic expression levels before and after percutaneous coronary intervention (PCI) in patients. Circulating miR-26a-1, miR-27a, miR-30d, miR-146a, miR-199a-1 and miR-423 were selected and validated in 31 AMI patients and 27 matched controls by quantitative real-time PCR (qPCR).

**Results:**

The expression levels of plasma miR-26a-1, miR-146a and miR-199a-1 were significantly increased in AMI patients. Receiver operating characteristic (ROC) analysis indicated that miR-26a-1, miR-146a and miR-199a-1 showed considerable diagnostic efficiency for predicting AMI. Furthermore, we demonstrated that the combination of miR-26a-1, miR-146a and miR-199a-1 facilitated AMI diagnosis.

**Conclusions:**

Our findings suggest that circulating miR-26a-1, miR-146a and miR-199a-1 have the potential to be used as biomarkers for AMI diagnosis.

**Electronic supplementary material:**

The online version of this article (10.1186/s10020-019-0086-1) contains supplementary material, which is available to authorized users.

## Introduction

Acute myocardial infarction (AMI) is a common disease with serious consequences in mortality and morbidity worldwide (Benjamin et al. 2017). A great number of patients with evolving myocardial infarction die before they reach hospital to receive medical treatment (Huikuri et al. 2001). In order to reduce mortality and improve prognosis of AMI, early and accurate diagnosis is needed for effective treatment (Shibata et al. 2015). The currently preferred diagnostic biomarkers for AMI are cardiac troponin I and T (cTnI and cTnT) (van der Linden et al. 2018; Roffi et al. 2016). However, false positive results with elevated cTn could associate with disease such as heart failure, chronic kidney diseases and sepsis, especially in elderly patients (Keller et al. 2009; Finsterer et al. 2007). In addition, as a marker of cell injury, cardiac troponin T (cTnT) does not peak until symptom onset. Therefore, additional biomarkers for AMI has been sought in order to improve diagnosis accuracy and also to help risk stratification.

MicroRNAs (miRNAs) are a class of short (~ 22 nucleotides) noncoding RNAs that play influential roles in post-transcriptional gene regulation (Bartel 2004). Numerous miRNAs have been identified to be involved in pathological process of cardiovascular diseases (Adachi et al. 2010; Cheng et al. 2010; Dong et al. 2009; Wang et al. 2015). Moreover, extracellular miRNAs are stable in body fluids such as plasma, serum and exosomes, may serve as reliable blood-based biomarkers (Mitchell et al. 2008; Cortez et al. 2011).

MiR-26a-1 is a member of the miR-26 family (miR-26a-1/26a-2/26b), which is located in chromosomes 3 (Han et al. 2012). Former studies have shown that miR-26a participates in cardiovascular disease by controlling endothelial cell growth, angiogenesis, and LV function post-MI (Icli et al. 2014). Studies also shown that miR-26a-1 inhibits apoptosis by targeting proapoptotic protein BAK1 in cells (Kulshreshtha et al. 2007). MiR-27a/b is expressed in endothelial cells and controls endothelial cell repulsion and angiogenesis by targeting semaphorin 6A (Urbich et al. 2011). Studies shown that miR-27a was related in regulating the cardiomyocytic apoptosis by targeting interleukin-10 pathway (Yeh et al. 2012). MiR-30d is expressed in cardiomyocytes and heart tissues and regulates cardiomyocyte pyroptosis by directly targeting foxo3a (Li et al. 2014). MiR-146a, which is located at the human chromosome 5q33, exhibited protective effect against cardiac ischemia/hypoxia-induced apoptosis (Huang et al. 2016), was also reported to be related to coronary artery disease (CAD) (Bao et al. 2015; Roldán et al. 2014). MiR-199a is principally expressed in cardiomyocytes, and the expression of miR-199a was previously reported to be up-regulated by 10-fold in hypertrophy hearts (Song et al. 2010; Rane et al. 2009). MiR-199a impairs autophagy and induces cardiac hypertrophy through mTOR activation (Li et al. 2015). MiR-423-5p could be released by thrombin-stimulated platelets during myocardial infarction and be taken up by endothelial cells (Gidlöf et al. 2013), and induces cell apoptosis in cardiomyocytes (Luo et al. 2015). These six miRNAs are engaged in the regulation of autophagy or apoptosis in cardiomyocytes during myocardial infarction. However, the diagnostic significance of these miRNAs in AMI needs further investigation. In this study, we aim to evaluate the plasma expression levels of miR-26a-1, miR-27a, miR-30d, miR-146a, miR-199a-1 and miR-423 in AMI patients before and after percutaneous coronary intervention (PCI) by comparing with health controls.

## Materials and methods

### Patients

Performance of the study was according to the principles of the Declaration of Helsinki and approved by the Ethics Committee of Affiliated Hospital of Qingdao University. All the data were collected for a period of 6 months (from Sep 2017 to Feb 2018). We collected blood samples from patients with AMI (*n* = 31) and healthy adults (*n* = 27) at Affiliated Hospital of Qingdao University after obtaining their written informed consent. For all participants, demographic and clinical characteristics were collected by trained interviewers using a standard structured questionnaire.

The AMI group consisted of 14 patients with STEMI and 17 patients with NSTEMI. All AMI patients underwent coronary angiography and PCI with chest pain onset less than 4 h duration. The inclusion criteria for AMI patients were based on the 2012 ESC/AHA/ACC guidelines (Thygesen et al. 2012). The criteria of AMI were as follows: (Benjamin et al. 2017) typical chest pain of greater than 30 min duration, (Huikuri et al. 2001) detection of increase of cardiac biomarker in serum, including cardiac troponin (cTn) and creatine kinase-MB (CK-MB), (Shibata et al. 2015) elevation or depression of the ST segment greater than 0.1 mV in two contiguous limb leaders, or 0.2 mV in two contiguous precordial leads or/and an abnormal Q wave. All patients met exclusion criteria, including previous history of cardiac diseases (MI, heart failure, cardiac arrhythmias, pacing or cardiomyopathy), known malignancy, renal insufficiency (serum creatinine concentration > 133 μmol/L), renal replacement therapy, surgery in the previous months.

Diabetes can be diagnosed on any of the following criteria (Aschner et al. 2014): 1. Fasting plasma glucose (FPG) ≥ 7.0 mmol/l (126 mg/dl). 2. Oral glucose (75 g) tolerance test (OGTT) with FPG ≥ 7.0 mmol/l (126 mg/dl) and/or 2-h plasma glucose ≥11.1 mmol/l (200 mg/dl). 3. Glycated haemoglobin (HbA1c) ≥ 6.5% /48 mmol/mol. 4. Random plasma glucose ≥11.1 mmol/l (200 mg/dl) in the presence of classical diabetes symptoms. 5. Asymptomatic individuals with a single abnormal test should have the test repeated to confirm the diagnosis unless the result is unequivocally elevated. There were 10 patients with type 2 diabetes mellitus in the AMI group, and they were treated with oral anti-diabetes medication by taking biguanides (3 patients), sulfonylureas (4 patients) and combination of biguanides and sulfonylureas (3 patients) respectively. In the control group, there were 3 patients with type 2 diabetes mellitus, and the oral anti-diabetes therapy was biguanides (2 patients) and sulfonylureas (1 patient) respectively.

Blood samples from study subjects were collected 1 h before PCI procedures and 1 h after PCI to investigate the expression level of plasma miRNAs. Healthy subjects without medical history of cardiovascular diseases were selected as controls, and they were matched by age, sex, and area of residence with the patients.

### Sample collection and storage

Vein blood sample (5 ml) was collected from each participant in EDTA-anticoagulant tubes (Sanli, Liuyang, China). All blood samples were centrifuged at 3000×g for 10 min at 4 °C, and the plasma supernatant was removed and transferred at − 80 °C until use.

### Plasma total RNA extraction

RNA was extracted from plasma by using TRIzol reagent (Life Technologies, Grand Island, NY, USA) according the instructions of the manufacturer. The plasma (250 μL) was thawed and mixed with 750 μL TRIzol, and then was shaken vigorously to ensure complete dissociation of nucleoprotein complexes. Each sample was supplemented with 5 μL aliquot of 50 pM synthetic *Caenorhabditis elegans* miR-39-3p (cel-miR-39-3p) after the addition of TRIzol to normalize miRNA expression as described previously (Niu et al. 2015; Fichtlscherer et al. 2010). After standing at room temperature (10 min), 200 μL chloroform was added to the mixture and shaken vigorously, the mixture was then centrifuged at 12,000×g for 10 min at 4 °C. The supernatant was transferred to a new tube, and 600 μL cold isopropanol was added. Glycogen (Thermo Scientific, Waltham, MA, USA) was also added to increase the RNA yield and the solution was precipitated at − 20 °C overnight. All samples were centrifuged at 12,000×g for 10 min at 4 °C again, and supernatants were discarded. Then, the RNA pellet was washed with 1 ml 75% ethanol. Finally, RNA was dissolved by adding DEPC H_2_O (10 μL) and stored at − 80 °C. The concentration and quality of RNA were measured by NanoDrop spectrophotometer (Thermo Fisher Scientific, Waltham, MA, USA).

### MicroRNA polyadenylation and reverse transcription

The cDNA was generated by using Mir-X™ miRNA First Strand Synthesis Kit (Clontech Laboratories, Mountain View, CA, USA) according to the manufacture’s protocol. The reaction was performed in a thermocycler with the following program: incubation at 37 °C for 1 h, then termination at 85 °C for 5 min to inactivate the enzymes. Finally, an aliquot of 90 μL double distilled water (ddH_2_O) was added to make a total volume of 100 μL in all.

### MicroRNA validation

The expression of the selected microRNA was determined by SYBR qPCR Kit (Takara, Dalian, China) according to the manufacture’s protocol with 2.0 μL cDNA as template. The primer sequences of miRNAs used in Real-Time PCR were listed in Supporting Information (Additional file 1: Table S1 and Figure S1-S3). The reaction was performed with the following program: 95 °C for 10 s, 40 cycles of 95 °C for 5 s, 60 °C for 20 s, and followed by the thermal denaturing step to generate the dissociation curves to verify amplification specificity. Cel-miR-39 was served as the normalization control, and data were analyzed by Bio-Rad CFX Manager software (Bio-Rad, CA, USA) to obtain miRNAs relative expression scores. Cycle threshold (Ct) values of each miRNA were normalized to cel-miR-39-3p, and the 2^−∆∆Ct^ method was used to analyzed the relative expression level of miRNA.

### Statistical analysis

For descriptive purposes data were presented as means ± standard deviations (SD) for quantitative variables. Mean values of quantitative variables were evaluated by Student’s *t-*test, or Mann-Whitney U test when Student’s *t-*test were not satisfied. For categorical variables, differences between cases and controls were analyzed by chi-square (χ^2^) test or Fisher’s exact test when necessary. The expression levels of miRNAs between patients and control subjects were compared by Student’s *t-*test. The associations of miRNA expression levels among each other and with clinical variables were analyzed by Spearman rank correlations. The combination among miRNAs were assessed using logistic regression. Receiver operating characteristic (ROC) curves and the area under the ROC curves (AUC) were performed to evaluate the diagnostic accuracy of the selected miRNAs using SigmaPlot 12.5 software (Systat Software, Inc., San Jose, CA, USA). SPSS 24.0 software (SPSS Inc., Chicago, IL, USA) was used to perform the statistical analyses, all statistical tests were two-tailed, and a value of *p* <  0.05 was considered statistically significant.

## Results

### Baseline characteristics of the study population

The baseline characteristics of 31 AMI patients and 27 control subjects were summarized in Table 1. The result showed that there were statistical differences between the control subjects group and AMI patients group. Clinical variables such as hypertension, triglycerides, CK-MB, Hs-TNT, and NT-proBNP were increased (*p* <  0.01) in the AMI patients compared with those without AMI (Table 1). The decreased level of Hs-cTNT after PCI reflecting the effectiveness of PCI (Additional file 1: Figure S1).

**Table 1:** Clinical characteristics of AMI patients and the control subjects

Variable	AMI group (*n* = 31)	Control group (*n* = 27)	*p*-value
Male/Female (n/n)^1^	25/6	19/8	0.362
Age (years)^2^	61.1 ± 10.0	60.1 ± 12.2	0.379
BMI (kg/m^2^)^2^	25.5 ± 2.1	24.8 ± 4.1	0.368
Smoking status			
Current smoker (%)^1^	58.1%	44.4%	0.188
Former smoker (%)^1^	9.7%	0	0.370
Never (%)^1^	32.2%	55.6%	0.074
Hypertension (%)^1^	71.0%	22.2%	< 0.001**
Diabetes (%)^1^	32.3%	11.1%	0.054
SBP (mmHg)^3^	129.4 ± 22.0	124.6 ± 11.9	0.258
DBP (mmHg)^3^	78.8 ± 12.8	71.9 ± 7.5	0.017*
Heart rate (beats/minutes)^3^	73.3 ± 12.2	77.3 ± 9.3	0.052
Killip class at admission ≥ II (%)^1^	32.3	0	0.001**
Blood glucose (mmol/L)^3^	6.7 ± 2.9	5.4 ± 2.0	0.720
Total cholesterol (mmol/L)^3^	4.7 ± 1.0	4.2 ± 1.5	0.119
Triglycerides (mmol/L)^3^	2.3 ± 3.0	1.1 ± 0.6	0.002**
HDL (mmol/L)^3^	1.1 ± 0.3	1.2 ± 0.5	0.307
LDL (mmol/L)^3^	2.7 ± 0.8	2.5 ± 1.1	0.293
WBC (× 10^9^/L)^3^	9.2 ± 3.0	7.7 ± 3.2	0.067
Cr (μmol/L)^3^	63.4 ± 14.0	70.0 ± 23.1	0.176
NT-proBNP (pg/ml)^3^	1497.5 ± 1667.4	77.2 ± 86.8	0.001**
CK-MB (U/L)^3^	47.5 ± 72.1	12.1 ± 8.9	< 0.001**
MYO (μg/L)^3^	55.4 ± 22.8	35.4 ± 10.7	0.250
Hs-cTNT (μg/L)^3^	2.08 ± 2.11	0.002 ± 0.000	< 0.001**
Concurrent medications			
ACE inhibitors (%)^1^	80.6%	0	< 0.001**
Beta-blockers (%)^1^	96.8%	0	< 0.001**
Nitrates (%)^1^	96.8%	3.7%	< 0.001**
Statins (%)^1^	100.0%	0	< 0.001**
Aspirins (%)^1^	100.0%	0	< 0.001**
Colpidogrel (%)^1^	41.9%	0	< 0.001**

*BMI* Body mass index, *DM* Diabetes mellitus, *SBP* systolic blood pressure, *DBP* diastolic blood pressure, *TC* total cholesterol, *TG* total triglycerides, *HDL* high-density lipoprotein, *LDL* low-density lipoprotein, *WBC* white blood cell, *Cr* creatinine, *CK-MB* Creatine Kinase-MB, *Hs-TNT* troponin T. Data are shown as the mean ± SD; ^1^Chi-square tests for the differences in the distribution frequencies between AMI patients and controls; ^2^Student’s *t* test for the differences between the AMI and control groups; ^3^Mann-Whitney U test for the differences between the AMI and control groups

### The expression patterns of circulating miRNAs by RT-qPCR

**Fig. 1:**
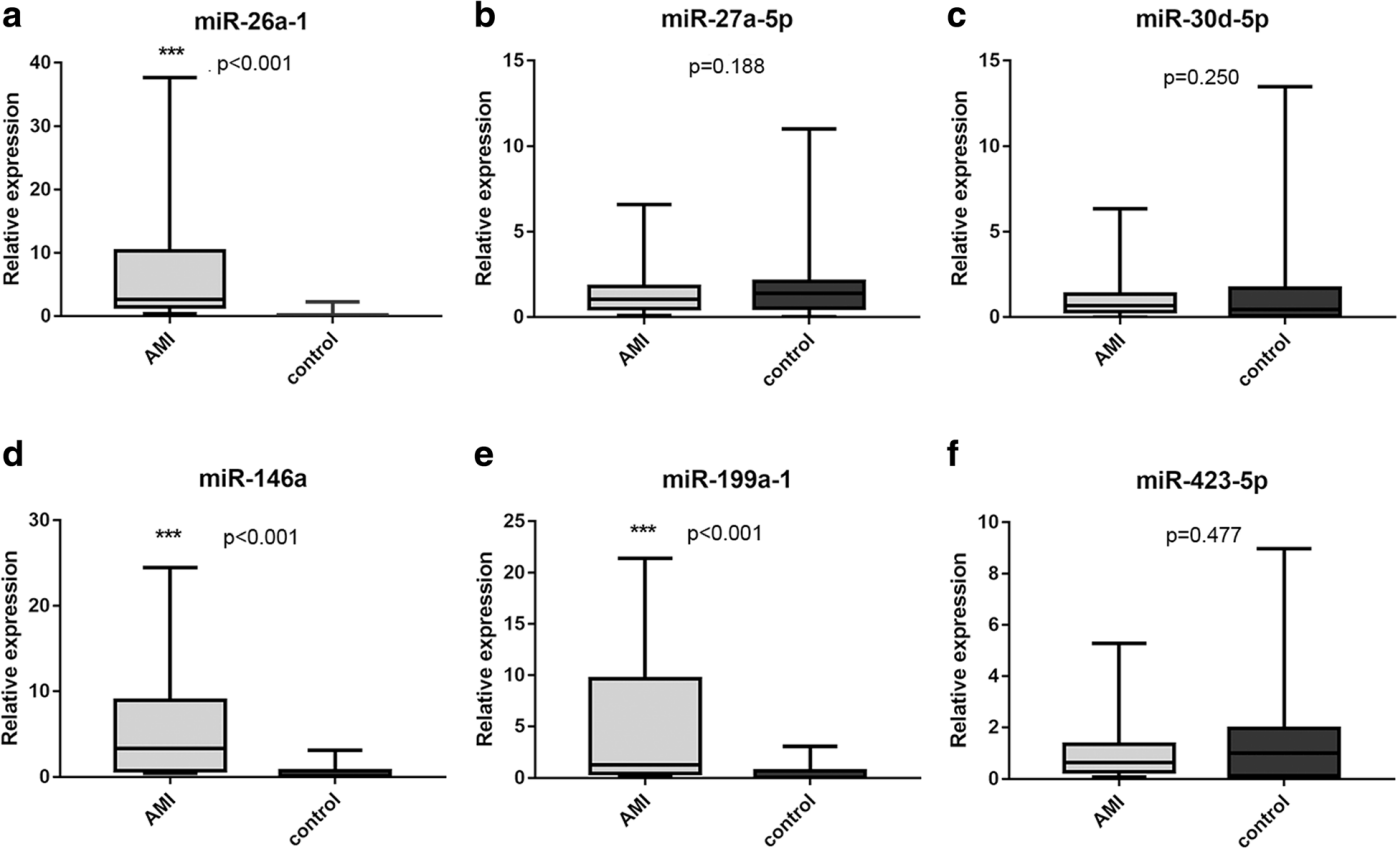
Plasma miRNA levels in the validation population. The box plots show the expression levels of miR-26a-1 (**a**), miR-27a (**b**), miR-30d (**c**), miR-146a (**d**), miR-199a-1 (**e**) and miR-423 (**f**) measured by quantitative real-time polymerase chain reaction (qPCR) in AMI patients (*n* = 31) and control subjects (*n* = 27). The relative miRNA expression levels were normalized to cel-miR-39 and calculated by –ΔΔCt. *p <  0.05, **p <  0.01, ***p <  0.001

**Fig. 2:**
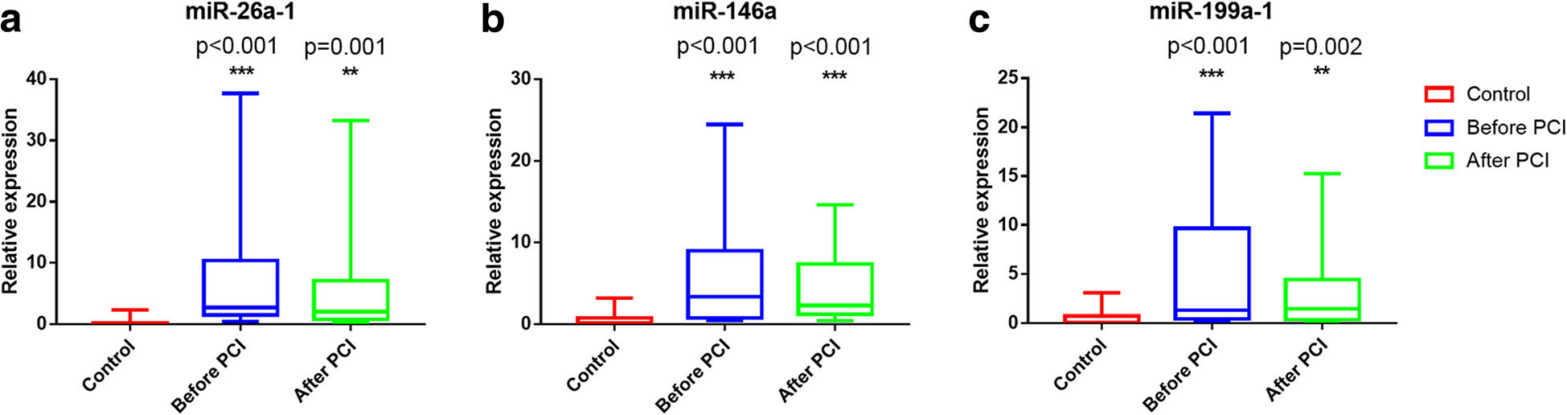
Plasma miRNA levels in the validation population before and after PCI. The box plots show the expression levels of miR-26a-1 (**a**), miR-146a (**b**) and miR-199a-1 (**c**)measured by qPCR in patients with AMI (including before and after PCI) (*n* = 31) and control subjects (*n* = 27). The relative miRNA expression levels were normalized to cel-miR-39 and calculated by –ΔΔCt. *p <  0.05, **p <  0.01, ***p <  0.001, compared with control subjects

Based on literature mining (Viereck and Thum 2017), several cardiovascular-related miRNA including miR-26a-1, miR-27a, miR-30d, miR-146a, miR-199a-1, and miR-423 were selected to investigate expression level in 31 AMI patients and 27 control subjects. These six miRNAs were chosen because they could be released into plasma during plaque rupture, thrombus formation, myocardial ischemia and reperfusion injury (necrosis and apoptosis). The result showed that the expression levels of miR-26a-1, miR-146a and miR-199a-1 significantly increased in AMI patients compared with control subjects (Fig. 1a, d and e). However, no significant difference was found in the expression levels of miR-27a, miR-30d and miR-423 between patients with AMI and control subjects (Fig. 1b, c and f). And then we further validated the expression of miR-26a-1, miR-146a and miR-199a-1before PCI and after PCI in whole population (including 31 AMI patients and 27 control subjects). The results showed that the expression levels of all three miRNAs were obviously increased in AMI patients both before and after PCI compared to control subjects (Fig. 2). The average expression level of circulating miR-26a-1 in AMI patients relative to the controls was increased by 8.10 ± 1.92 before PCI and by 5.60 ± 1.46 after PCI (Fig. 2a). Likewise, the circulating level of miR-146a was increased by 6.18 ± 1.20 before PCI and by 4.33 ± 0.73 after PCI (Fig. 2b). Circulating miR-199a-1 was increased by 4.71 ± 1.00 before PCI and by 2.93 ± 0.65 after PCI (Fig. 2c). These results also showed a decrease of miRNA level after PCI compared with that before PCI.

**Fig. 3:**
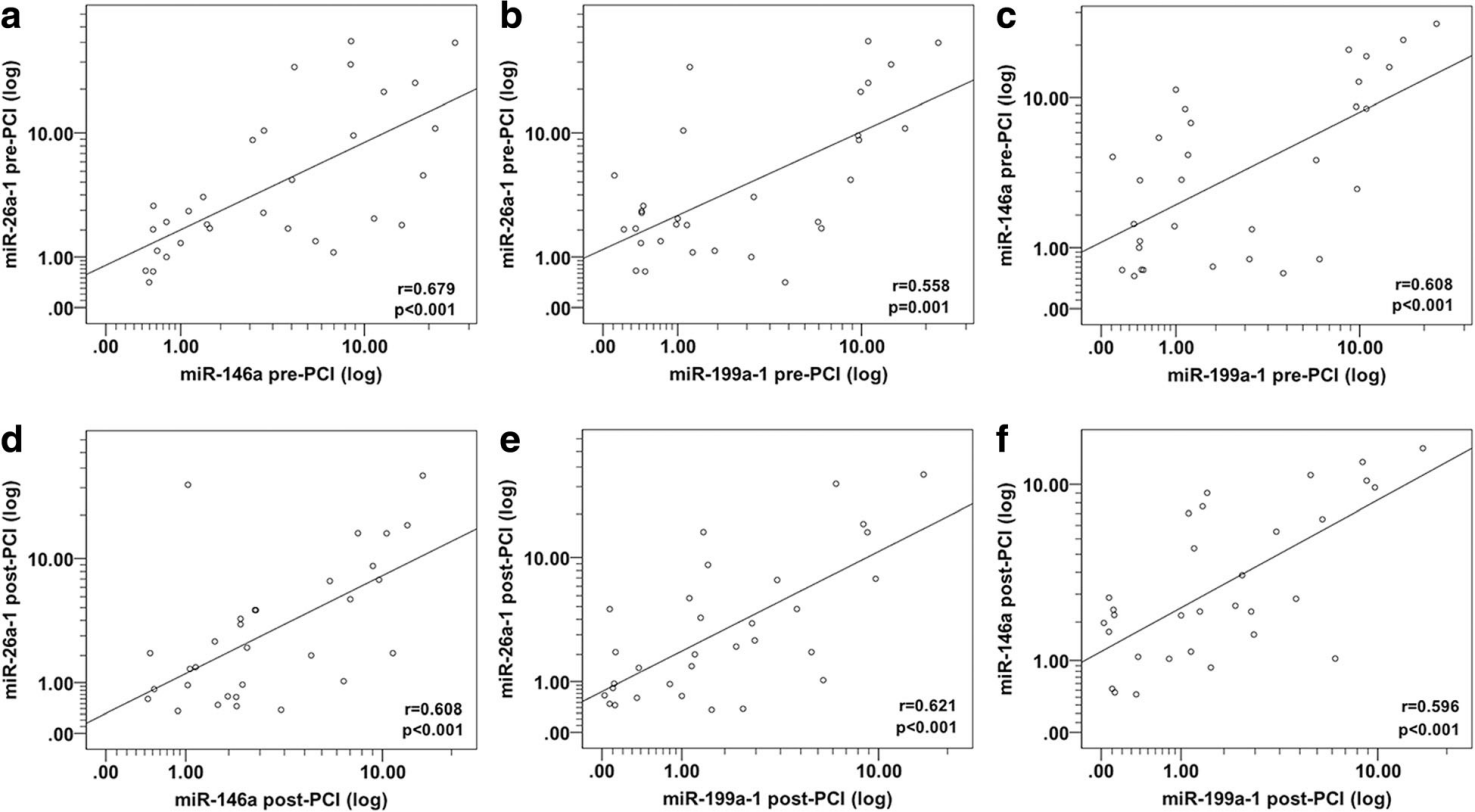
Spearman correlations between the circulating miRNA in patients with AMI before and after PCI. The scatter plots show the marked correlation in the expression values between miRNAs: **a** between miR-26a-1 pre-PCI and miR-146a pre-PCI, **b** between miR-26a-1 pre-PCI and miR-199a-1 pre-PCI, **c** between miR-146a pre-PCI and miR-199a-1 pre-PCI, **d** between miR-26a-1 post-PCI and miR-146a post-PCI, **e** between miR-26a-1 post-PCI and miR-199a-1 post-PCI, **f** between miR-146a post-PCI and miR-199a-1post-PCI, in the population with AMI (*n* = 31)

**Fig. 4:**
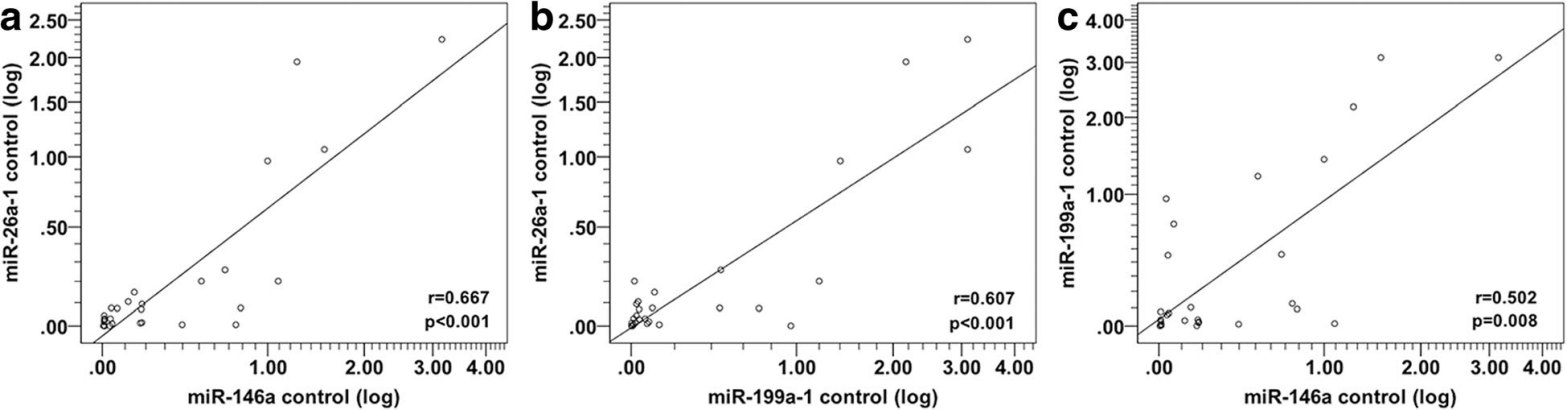
Spearman correlations between circulating miRNA in control subjects. The scatter plots show the marked correlation in the expression values between **a** miR-26a-1 and miR-146a and **b** miR-199a-1, and **c** between miR-146a and miR-199a-1 in the control population (*n* = 27)

We performed Spearman correlation analysis of miRNA expression to ascertain whether these miRNAs are related or not to each other. Interestingly, we found a striking correlation between miR-26a-1, miR-146a and miR-199a-1 expression levels in the group of patients with AMI before PCI (Fig. 3a, b and c), after PCI (Fig. 3d, e and f), and also in the control group (Fig. 4a–c).

### The correlation and multivariate analysis

**Fig. 5:**
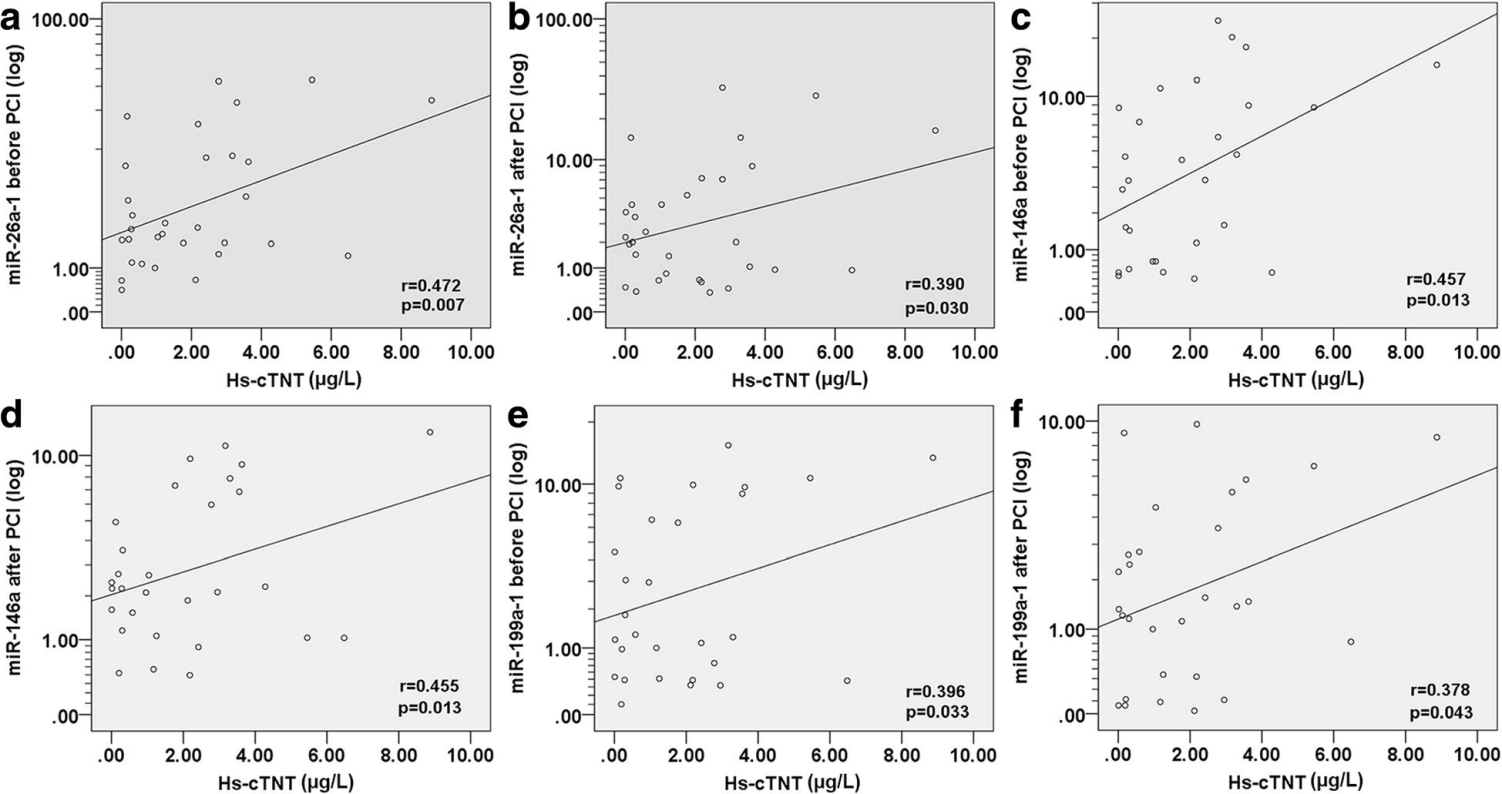
Correlations between circulating miRNAs and Hs-cTNT in AMI patients. The scatter plots show the correlation between Hs-cTNT and **a** miR-26a-1 pre-PCI and **b** miR-26a-1 post-PCI and **c** miR-146a pre-PCI and **d** miR-146a post-PCI and **e** miR-199a-1 pre-PCI and **f** miR-199a-1 post-PCI, in the population (*n* = 31) with AMI

The correlation coefficients between the levels of miR-26a-1, miR-146a and miR-199a-1 and metabolic parameters or cardiovascular risk factors were analyzed (Table 2). A significant positive correlation of miR-26a-1 (*r* = 0.543, *p* = 0.002), miR-146a (*r* = 0.510, *p* = 0.003) and miR-199a-1 (*r* = 0.390, *p* = 0.030) level was found with NT-proBNP before PCI (Table 2). NT-proBNP was also significantly correlated with miR-26a-1 (*r* = 0.357, *p* = 0.048) and miR-199a-1 (*r* = 0.445, *p* = 0.012) level in patients with AMI after PCI (Table 2). Univariate logistic regression analysis revealed that miR-26a-1 (*p* = 0.001), miR-146a (*p* = 0.005) and miR-199a-1 (*p* = 0.009) before PCI were significantly associated with the risk of AMI (Table 3). The significant association between the risk of AMI and miR-26a-1 (*p* = 0.001), miR-146a (*p* = 0.005) and miR-199a-1 (*p* = 0.002) after PCI were also observed (Table 3). These miRNAs were placed into a forward, stepwise, multivariate logistic regression model that includes hypertension, smoking, LDL, cholesterol and total triglycerides. After adjustment, the expression of miR-26a-1, miR-146a, and miR-199a-1 were still significantly associated with the risk of AMI (Table 3). We investigated the correlation coefficients between Hs-cTNT and miRNAs. The results indicated that the expression levels before PCI of miR-26a-1 (*r* = 0.472, *p* = 0.007), miR-146a (*r* = 0.457, *p* = 0.013) and miR-199a-1 (*r* = 0.396, *p* = 0.033) exhibited a significantly positive correlation with Hs-cTNT respectively (Fig. 5a, c, e). The expression levels after PCI of miR-26a-1 (*r* = 0.390, *p* = 0.030), miR-146a (*r* = 0.455, *p* = 0.013) and miR-199a-1 (*r* = 0.378, *p* = 0.043) were also significantly positive correlated with Hs-cTNT (Fig. 5b, d, f). We also examined the relationship between left ventricular ejection fraction (LVEF) and miRNAs. The results showed that the expression levels of miRNAs before PCI and after PCI exhibited no significant correlation with LVEF (Additional file 1: Figure S2a–f).

**Table 2:** Relationships between miR-26a-1, miR-146a and miR-199a-1 and classical cardiovascular risk factors in patients with AMI and control subjects

Variable		AMI before PCI (*n* = 31)			AMI after PCI (*n* = 31)			Controls (*n* = 27)		
		miR-26a-1	miR-146a	miR-199a-1	miR-26a-1	miR-146a	miR-199a-1	miR-26a-1	miR-146a	miR-199a-1
Age (years)	*r*=	−0.150	−0.026	0.047	−0.211	0.123	0.083	0.249	0.319	0.011
	*p*=	0.421	0.89	0.804	0.255	0.509	0.657	0.211	0.105	0.955
BMI (kg/m^2^)	*r*=	0.188	0.112	0.179	0.194	0.112	0.226	−0.117	− 0.370	− 0.050
	*p*=	0.310	0.550	0.336	0.295	0.550	0.222	0.560	0.856	0.803
SBP (mmHg)	*r*=	−0.020	0.063	−0.114	−0.061	−0.095	−0.026	0.021	−0.196	−0.283
	*p*=	0.913	0.735	0.542	0.745	0.610	0.891	0.916	0.326	0.153
DBP (mmHg)	*r*=	−0.025	0.112	−0.202	−0.226	−0.151	−0.172	−0.206	−0.368	−0.124
	*p*=	0.895	0.550	0.275	0.221	0.418	0.355	0.303	0.059	0.538
HP (cmp)	*r*=	0.035	0.055	0.069	0.015	−0.077	−0.190	0.071	0.096	0.279
	*p*=	0.851	0.767	0.713	0.935	0.682	0.306	0.723	0.633	0.159
GLU (mmol/L)	*r*=	−0.150	0.022	0.146	−0.181	−0.041	0.068	−0.126	−0.190	−0.147
	*p*=	0.421	0.906	0.435	0.330	0.829	0.716	0.533	0.343	0.464
Tg (mmol/L)	*r*=	0.028	0.066	−0.188	−0.007	0.400	−0.031	−0.315	−0.325	0.240
	*p*=	0.881	0.726	0.311	0.969	0.832	0.868	0.110	0.098	0.228
Tc (mmol/L)	*r*=	**−0.413**	−0.143	−0.225	−0.115	− 0.161	− 0.118	− 0.074	− 0.264	− 0.136
	*p*=	**0.021**	0.444	0.166	0.540	0.387	0.527	0.713	0.184	0.500
HDL-C (mmol/L)	*r*=	0.145	−0.040	−0.099	−0.033	−0.278	−0.062	0.160	−0.207	0.041
	*p*=	0.437	0.831	0.598	0.861	0.130	0.742	0.425	0.299	0.839
LDL-C (mmol/L)	*r*=	−0.246	−0.046	−0.242	−0.074	−0.191	−0.012	−0.201	−0.297	−0.104
	*p*=	0.182	0.804	0.189	0.693	0.303	0.948	0.315	0.132	0.605
WBC (*10^9/L)	*r*=	−0.224	−0.255	−0.166	−0.060	−0.040	−0.212	0.051	0.321	0.345
	*p*=	0.235	0.174	0.381	0.752	0.835	0.261	0.801	0.103	0.078
Cr (μmol/L)	*r*=	−0.110	−0.015	−0.062	−0.104	−0.046	−0.067	0.123	0.102	0.095
	*p*=	0.563	0.936	0.744	0.584	0.808	0.727	0.559	0.628	0.650
NT-proBNP (ng/mL)	*r*=	**0.543**	**0.510**	**0.390**	**0.357**	0.277	**0.445**	–	–	–
	*p*=	**0.002**	**0.003**	**0.030**	**0.048**	0.132	**0.012**	–	–	–
CK-MB (μg/L)	*r*=	0.008	−0.070	−0.060	−0.131	0.027	−0.096	−0.096	−0.199	−0.312
	*p*=	0.965	0.715	0.753	0.489	0.887	0.615	0.655	0.351	0.138
Hs-cTNT (μg/L)	*r*=	**0.472**	**0.457**	**0.396**	**0.390**	**0.455**	**0.378**	–	–	–
	*p*=	**0.007**	**0.013**	**0.033**	**0.030**	**0.013**	**0.043**	–	–	–

*r* = Spearman rank correlation coefficients, *p* = *p* value. Values with statistical significance were indicated in bold. *BMI* Body mass index, *DM* Diabetes mellitus, *SBP* systolic blood pressure, *DBP* diastolic blood pressure, *TC* total cholesterol, *TG* total triglycerides, *HDL* high-density lipoprotein, *LDL* low-density lipoprotein, *WBC* white blood cell, *Cr* creatinine, *CK-MB* Creatine Kinase-MB, *Hs-TNT* troponin T

**Table 3:** Multivariate logistic regression analysis for the risk of AMI

	Univariate logistic regression			Multivariate logistic regression		
	OR	95% CI	*p*-value	OR	95% CI	*p*-value
miR-26a-1 pre-PCI	16.573	3.405–80.670	0.001	17.063	2.619–111.176	0.003
miR-26a-1 post-PCI	16.168	3.323–78.656	0.001	26.549	2.549–276.556	0.006
miR-146a pre-PCI	12.787	2.137–76.509	0.005	12.133	1.706–86.296	0.013
miR-146a post-PCI	5.254	1.657–16.658	0.005	5.864	1.618–21.250	0.007
miR-199a-1 pre-PCI	8.287	1.679–40.900	0.009	4.949	1.138–21.528	0.033
miR-199a-1 post-PCI	13.389	2.496–71.815	0.002	11.155	1.955–63.661	0.007

The model included hypertension, smoking, LDL, cholesterol and total triglycerides. *CI* confidence interval, *OR* odds ratio. OR were given for variation of one unit of miRNA

### The diagnostic accuracy of the candidate miRNA in AMI

**Fig. 6:**
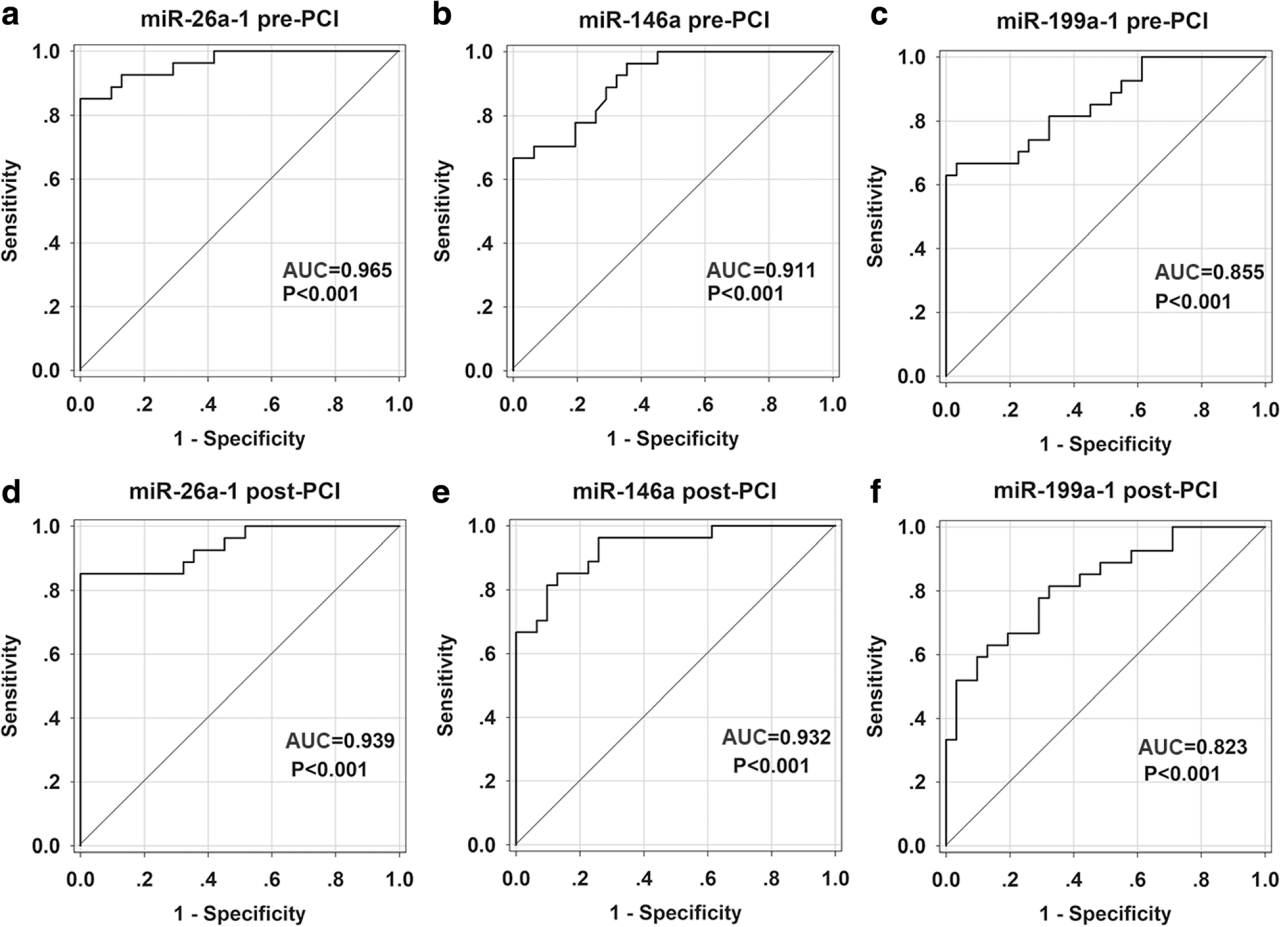
Receiver operating characteristic (ROC) curves analysis of miR-26a-1, miR-146a and miR-199a-1 for predicting AMI. The areas under the curves (AUC) are 0.965 (95% CI: 0.924–1.007, *p* < 0.001) for miR-26a-1 pre-PCI (**a**), 0.939 (95% CI: 0.877–1.001, *p* < 0.001) for miR-26a-1 post-PCI (**d**), 0.911 (95% CI: 0.841–0.981, *p* < 0.001) for miR-146a pre-PCI (**b**), 0.932 (95% CI: 0.870–0.994, *p* < 0.001) for miR-146a post-PCI (**e**), 0.855 (95% CI: 0.759–0.952, *p* < 0.001) for miR-199a-1 pre-PCI (**c**), 0.823 (95% CI: 0.717–0.929, *p* < 0.001) for miR-199a-1 post-PCI (**f**). CI, confidence interval

**Fig. 7:**
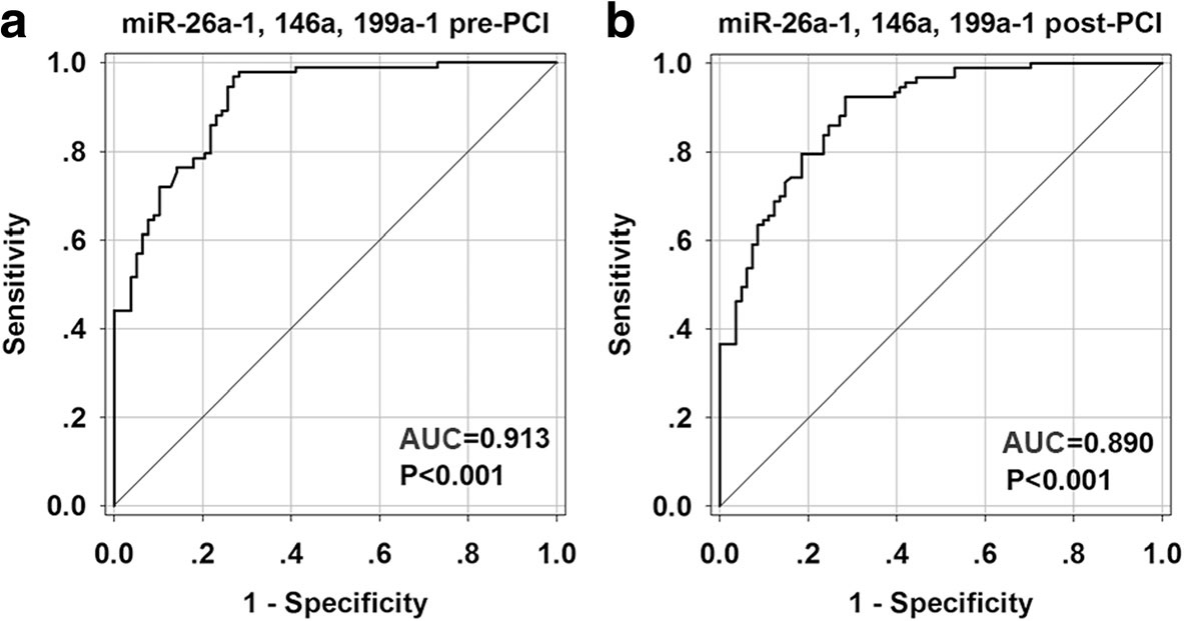
Receiver operating characteristic (ROC) curves analysis of the combination of miR-26a-1, miR-146a, miR-199a-1 for predicting AMI. The areas under the curves (AUC) are 0.913 (95% CI: 0.872–0.954, *p* < 0.001) for the combination of miR-26a-1, miR-146a, miR-199a-1 pre-PCI (**a**) and 0.890 (95% CI: 0.843–0.937, *p* < 0.001) for the combination of miR-26a-1, miR-146a, miR-199a-1 post-PCI (**b**). CI, confidence interval

To investigate the diagnostic accuracy of these circulating miRNA, a ROC curve analysis was performed. As shown in Fig. 6, the area under the curve (AUC) before PCI was 0.965 (95% CI: 0.924–1.007, *p* <  0.001) with cut-off value of 0.852 (100% sensitivity, 85.2% specificity) for miR-26a-1 (Fig. 6a), 0.911 (95% CI: 0.841–0.981, *p* <  0.001) with cut-off value of 0.667 (100% sensitivity, 66.7% specificity) for miR-146a (Fig. 6b), 0.855 (95% CI: 0.759–0.952, *p* <  0.001) with cut-off value of 0.634 (96.8% sensitivity, 66.7% specificity) for miR-199a-1 (Fig. 6c). We also investigated the AUC value after PCI, which was 0.939 (95% CI: 0.877–1.001, *p* <  0.001) with cut-off value of 0.852 (100% sensitivity, 85.2% specificity) for miR-26a-1 (Fig. 6d), 0.932 (95% CI: 0.870–0.994, *p* <  0.001) with cut-off value of 0.723 (87.1% sensitivity, 85.2% specificity) for miR-146a (Fig. 6e), 0.823 (95% CI: 0.717–0.929, *p* <  0.001) with cut-off value of 0.501 (87.1% sensitivity, 63% specificity) for miR-199a-1 (Fig. 6f). To further evaluate the diagnostic effects of these three miRNAs, the ROC curves of combination of miR-26a-1, miR-146a and miR-199a-1 were constructed (Fig. 7). The results showed a high AUC value of 0.913 (95% CI: 0.872–0.954, *p* <  0.001) with cut-off value of 0.695 (97.8% sensitivity, 71.6% specificity) before PCI (Fig. 7a) and 0.890 (95% CI: 0.843–0.937, *p* <  0.001) with cut-off value of 0.640 (92.5% sensitivity, 71.6% specificity) after PCI (Fig. 7b). The ROC curve analysis was also performed to compare the diagnostic accuracy of Hs-cTNT and miRNAs. As shown in Additional file 1: Figure S3, the AUC value was 0.897 (95% CI: 0.817–0.978, *p* <  0.001) with cut-off value of 0.686 (87.1% sensitivity, 81.5% specificity) for Hs-cTNT.

## Discussion

An early and sensitive diagnosis of AMI may improve the survival rate of patients. In order to improve initial diagnosis and also to help risk management, additional markers, including circulating miRNAs, have been sought (McCann et al. 2008). Based on their tissue-specific expression pattern, repaid release into the circulation and remarkable stability in plasma, circulating miRNAs showed their potential as biomarkers for cardiovascular diseases (Viereck and Thum 2017; Fichtlscherer et al. 2011). Monitoring miRNA by RT-qPCR may be a potential new approach for rapid and non-invasive diagnosis of disease (Poller et al. 2017).

In this study, we demonstrated for the first time that miR-26a-1, miR-146a and miR-199a-1 may serve as candidate diagnostic biomarkers for AMI, allowing to distinguish patients with AMI from non-AMI control subjects. The present study showed that the expression levels of circulating miR-26a-1, miR-146a and miR-199a-1 were significantly up-regulated in the plasma of patients with AMI compared to control subjects. Multivariate analysis showed that high levels of circulating miR-26a-1, miR-146a and miR-199a-1 were associated with AMI (including events before and after PCI). Further analysis confirmed that miR-26a-1, miR-146a and miR-199a-1 are related to the diagnosis of AMI. The receiver operating curve (ROC) analysis further revealed that miR-26a-1, miR-146a and miR-199a-1 may be potential markers for AMI. Among the miRNAs in this study, miR-26a-1 showed the most accurate diagnosing result with the highest AUC values before and after PCI. The AUC values of miR-26a-1 both before and after PCI (0.956 and 0.939 respectively) were very similar to the AUC of cardiac-enriched miR-208 (AUC = 0.965) reported previously (Wang et al. 2010). Furthermore, miR-26a-1 exhibited well positive correlation with Hs-cTNT both before and after PCI. These results demonstrated the great value in clinical implication of miR-26a-1. The AUC values of miR-26a-1 and miR-146a were higher than that of Hs-cTNT, so we hypothesized that miRNAs could add incremental diagnostic value to Hs-cTNT. The combination of the three miRNAs showed the high AUC value before PCI and after PCI, indicating that the combination of miRNAs also has higher accuracy in diagnosing of AMI. Sex, age and cardiovascular risk factors may contribute to changes of miRNA levels between patients with cardiovascular disease and control subjects (Goretti et al. 2014). In our research, the studied population was matched on sex, age and risk factors such as diabetes, obesity, smoking habit, and renal dysfunction. The levels of these three miRNAs were observed increased in in the early stage of AMI (within 4 h of onset of symptoms). MiRNAs changes could be detected at the early stage of AMI was considered as the advantages over traditional diagnostic marker (Creemers et al. 2012). Interestingly, the levels of miRNA after PCI were decreased compared with that before PCI, and this was in accordance with the change of Hs-cTNT. These results support the significance of these miRNAs as biomarker in AMI.

Prior studies have shown that necrosis and apoptosis result in myocardial ischemia or reperfusion injury, leading to pathological remodeling of the heart (McCully et al. 2004). miR-26a-1 is a member of the miR-26 family (miR-26a-1/26a-2/26b), which is located in chromosomes 3 (Han et al. 2012). MiR-26 family is enriched in the heart, its expression is increased early post-MI in the ischemic zone, and neutralization of miR-26a improved left ventricular (LV) function by decreasing cardiomyocyte apoptosis and increasing angiogenesis (Leeper et al. 2011). MiR-146a is enriched in cardiosphere-derived cells (CDCs) circulating exosomes (Ibrahim Ahmed et al. 2014), and significantly up-regulated in human atherosclerotic plaques (Raitoharju et al. 2011). MiR-146a plays an important role in myocardial infarction pathology by targeting two toll-like receptors (Irak1 and Traf6) (Huang et al. 2012; Wang et al. 2013). Various factors can affect the inflammatory process of atherosclerosis, including necrosis and apoptosis. miR-146a has been reported to be involved in the inflammatory process of atherosclerosis, by targeting interleukin-1 receptor-associated kinase 1 (IRAK-1) and TNF receptor-associated factor 6 (TRAF-6) (Bao et al. 2015; Ramkaran et al. 2014). Previous studies have demonstrated that miR-199a-1 is predominantly expressed in cardiomyocytes (Song et al. 2010; Rane et al. 2009). MiR-199a is essential for the maintenance of cardiomyocytes cell size (Song et al. 2010). The expression of miR-199a was up-regulated in cardiac hypertrophy and failure studies, and overexpression of miR-199a led to elongated myocytes (Van Rooij et al. 2006). Altogether these results we propose that the up-regulation of these miRNAs plays a critical role in the pathogenesis of AMI, such as ischemia-reperfusion injury (necrosis and apoptosis). This could explain the association between the up-regulation of these microRNAs and the diagnosis in the early phase of AMI.

Our study showed the significance of increased miR-26a-1, miR-146a and miR-199a-1 may be considered as potential candidate biomarkers for early diagnosis of AMI. This study is based on cohort of patients with AMI, and a double-blind and randomised clinical study with large cohort of subjects without a diagnosis of AMI at the start of study will be needed in the future to confirm the diagnostic value of these miRNAs. Therefore, to make these microRNAs reach bedside, clinical trials with large sample sizes and long-term follow-ups will be performed.

## Conclusion

We investigated the dynamic expressions of circulating miR-26a-1, miR-146a and miR-199a-1 in the patients with AMI (before and after PCI) for the first time. Our results proved that circulating miR-26a-1, miR-146a and miR-199a-1 may be considered as promising biomarkers for early diagnosis of AMI by using blood based non-invasive methods. The unique signature of circulating miRNA in AMI patients suggests that plasma miR-26a-1, miR-146a and miR-199a-1 may provide useful information to illustrate the mechanism underlying the pathogenesis of AMI.

## Additional file

Additional file 1: **Table S1.** Primer sequences of miRNAs. **Figure S1.** High-sensitivity troponin T (hs-cTnT) levels among AMI patients. The box plots show the level of hs-TnT. Hs-TnT level was evaluated 1 h before PCI procedures and 1 h after PCI in the population with AMI (*n* = 31). **Figure S2.** Correlations between circulating miRNAs and LVEF in AMI patients. The scatter plots show the correlation between LVEF and (a) miR-26a-1 pre-PCI and (b) miR-146a pre-PCI and (c) miR-199a-1 pre-PCI and (d) miR-26a-1 post-PCI and (e) miR-146a post-PCI and (f) miR-199a-1 post-PCI, in the population (*n* = 31) with AMI. **Figure S3.** Receiver operating characteristic (ROC) curves analysis of Hs-cTNT for predicting AMI. The areas under the curves (AUC) are 0.897 (95% CI: 0.817–0.978, *p* <  0.001) for Hs-cTNT. CI, confidence interval (DOCX 5449 kb)

## Abbreviations

ACC: American College of Cardiology
AHA: American Heart Association
AMI: Acute myocardial infarction
AUC: Area under the ROC curves
CAD: Coronary artery disease
CK-MB: Creatine kinase-MB
cTnI: Cardiac troponin I
cTnT: Cardiac troponin T
DEPC: Diethypyrocarbonate
EDTA: Ethylene Diamine Tetraacetic Acid
ESC: European Society of Cardiology
Hs-cTnT: High-sensitivity troponin T
LV: Left ventricle
MI: Myocardial infarction
miRNA: microRNA
NSTEMI: Non-ST-segment elevation myocardial infarction
NT-proBNP: N-terminal pro-B-type natriuretic peptide
PCI: Percutaneous coronary intervention
Real-Time PCR: Real-time polymerase chain reaction
ROC: Receiver operating characteristic
RT-qPCR: Reverse transcription-quantitative real-time PCR
STEMI: ST-segment elevation myocardial infarction
