# Supplementary material for: Circulating miR-26a-1, miR-146a and miR-199a-1 are potential candidate biomarkers for acute myocardial infarction
Source: Mol Med. 2019 May 15;25:18. doi: 10.1186/s10020-019-0086-1 (PMC6521554; doi:10.1186/s10020-019-0086-1)
Supplement: Supplementary file 1 — Table S1. Primer sequences of miRNAs. Figure S1. High-sensitivity troponin T (hs-cTnT) levels among AMI patients. The box plots show the level of hs-TnT. Hs-TnT level was evaluated 1 h before PCI procedures and 1 h after PCI in the population with AMI (n = 31). Figure S2. Correlations between circulating miRNAs and LVEF in AMI patients. The scatter plots show the correlation between LVEF and (a) miR-26a-1 pre-PCI and (b) miR-146a pre-PCI and (c) miR-199a-1 pre-PCI and (d) miR-26a-1 post-PCI and (e) miR-146a post-PCI and (f) miR-199a-1 post-PCI, in the population (n = 31) with AMI. Figure S3. Receiver operating characteristic (ROC) curves analysis of Hs-cTNT for predicting AMI. The areas under the curves (AUC) are 0.897 (95% CI: 0.817–0.978, p < 0.001) for Hs-cTNT. CI, confidence interval (DOCX 5449 kb) [file 10020_2019_86_MOESM1_ESM.docx]

**Circulating miR-26a-1, miR-146a and miR-199a-1 are Potential Candidate Biomarkers for Acute Myocardial Infarction**

Sheng Xue^1^*, Wenjie Zhu^2^*, Dacheng Liu^2^, Zhe Su^2^, Liwei Zhang^2^, Qing Chang^2^ & Peifeng Li^1^

^1^ Institute for Translational Medicine, College of Medicine, Qingdao University, Qingdao, 266021, China.

^2^ Affiliated Hospital of Qingdao University, Qingdao University, Qingdao, 266003 China.

Correspondence and requests of materials should be addressed to:

SX (shengxue198@126.com), QC (changqing20671@163.com) and PL (peifli@qdu.edu.cn)

*These authors contributed equally to this work.

**Table S1.** Primer sequences of miRNAs.

| microRNAs | Primer |
| --- | --- |
| miR-26a-1 | F Primer: TTCAAGTAATCCAGGATAGGCT |
| miR-27a-5p | F Primer: AGGGCTTAGCTGCTTGTGAGCA |
| miR-30d-5p | F Primer: TGTAAACATCCCCGACTGGAAG |
| miR-146a | F Primer: TGAGAACTGAATTCCATGGGTT |
| miR-199a-1 | F Primer: CCCAGTGTTCAGACTACCTGTTC |
| miR-423-5p | F Primer: TGAGGGGCAGAGAGCGAGACTTT |
| cel-miR-39 | F Primer: TCACCGGGTGTAAATCAGCTTG |
| Unified reverse primer | R Primer: TGGTGTCGTGGAGTCG |

**Figure S1**

**
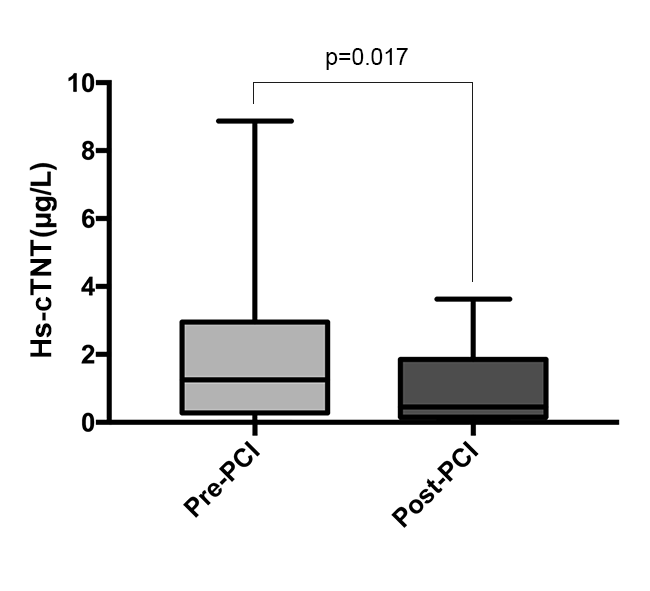
**

**Figure S1. High-sensitivity troponin T (hs-cTnT) levels among AMI patients.** The box plots show the level of hs-TnT. Hs-TnT level was evaluated 1 h before PCI procedures and 1 h after PCI in the population with AMI (n=31).

**Figure S2**


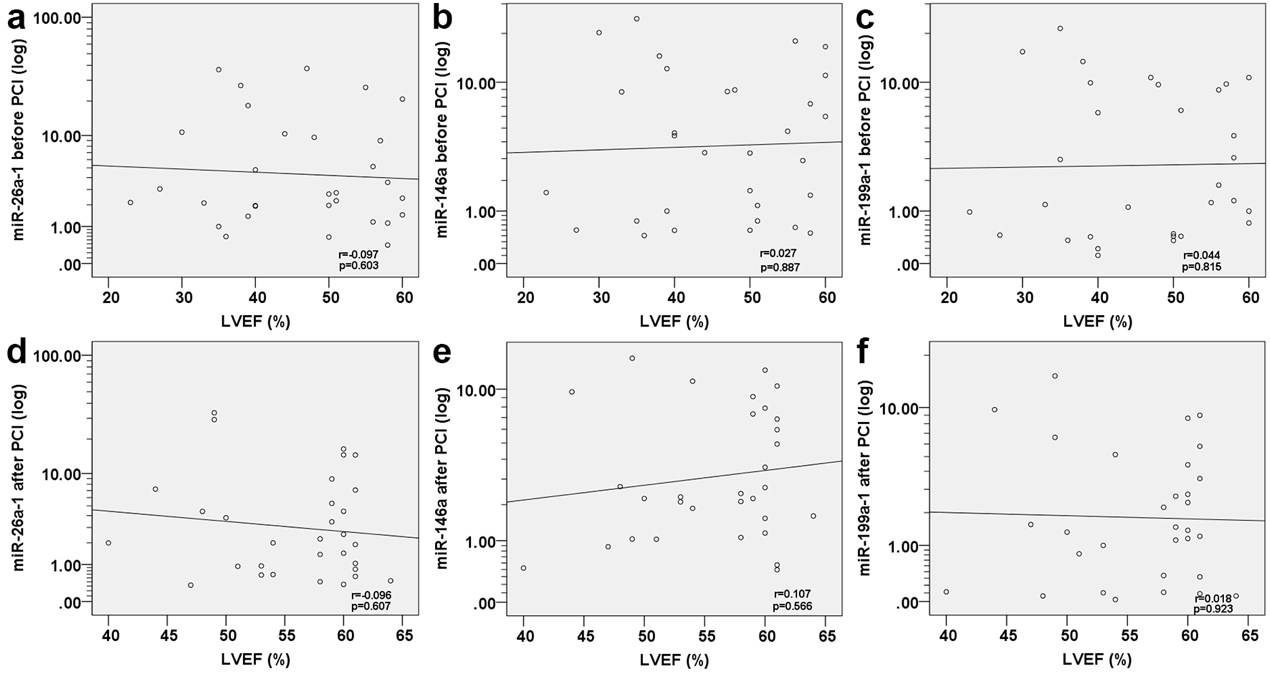


**Figure S2. Correlations between circulating miRNAs and LVEF in AMI patients.** The scatter plots show the correlation between LVEF and (a) miR-26a-1 pre-PCI and (b) miR-146a pre-PCI and (c) miR-199a-1 pre-PCI and (d) miR-26a-1 post-PCI and (e) miR-146a post-PCI and (f) miR-199a-1 post-PCI, in the population (n=31) with AMI.

**Figure S3**

**
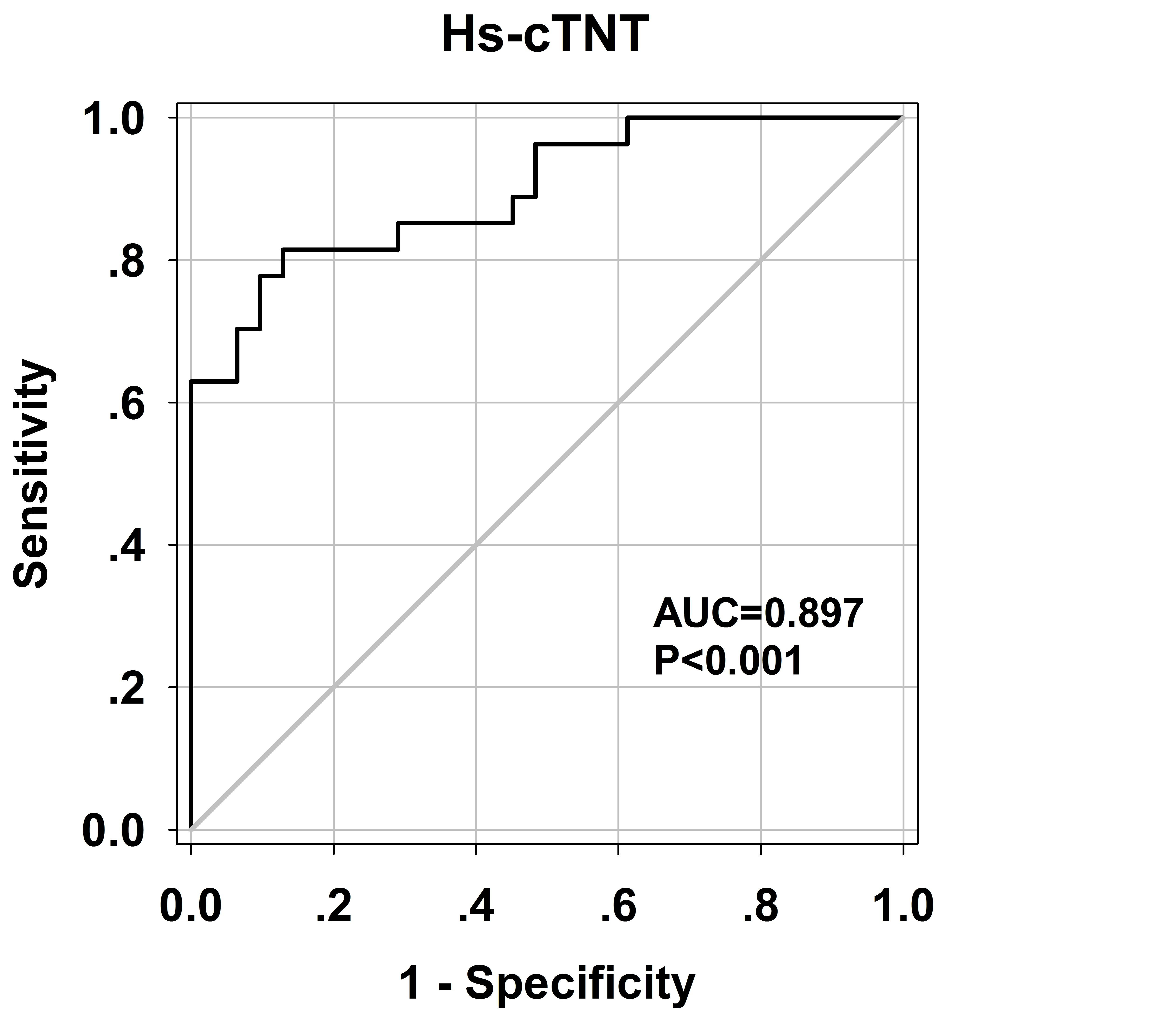
**

**Figure S3. Receiver operating characteristic (ROC) curves analysis of Hs-cTNT for predicting AMI.** The areas under the curves (AUC) are 0.897 (95% CI: 0.817–0.978, p<0.001) for Hs-cTNT. CI, confidence interval.
